# Evolving Treatment of Advanced Hepatocellular Carcinoma in the Asia–Pacific Region: A Review and Multidisciplinary Expert Opinion

**DOI:** 10.3390/cancers13112626

**Published:** 2021-05-27

**Authors:** Sadahisa Ogasawara, Su-Pin Choo, Jiang-Tao Li, Changhoon Yoo, Bruce Wang, Dee Lee, Pierce K. H. Chow

**Affiliations:** 1 Department of Gastroenterology, Graduate School of Medicine, Chiba University, 1-8-1 Inohana, Chuo-ku, Chiba 260-8670, Japan; ogasawaras@chiba-u.jp; 2 Curie Oncology, 38 Irrawaddy Road #08-21/29, Mount Elizabeth Novena Specialist Centre, Singapore 329563, Singapore; choo.su.pin@singhealth.com.sg; 3 Department of Surgery, Second Affiliated Hospital, Zhejiang University School of Medicine, 88 Jiefang Street, Hangzhou 310009, China; zrljt@zju.edu.cn; 4 Department of Oncology, Asan Medical Center and University of Ulsan College of Medicine, 88 Olympic-ro 43-gil, Songpa-gu, Seoul 05505, Korea; yooc@amc.seoul.kr; 5 Elysia Group Ltd., Xiamen Street, Lane 113, No 17-1, Floor 2, Taipei 10082, Taiwan; bruce.wang@elysiagroup.com; 6 Inno Community Development Organisation, Dezheng South Business Center, 57 Dezheng S. Road, Yuexiu District, Guangzhou 510000, China; dee.lee@theinno.org; 7 National Cancer Centre Singapore and Duke-NUS Medical School, 11 Hospital Crescent, Singapore 169610, Singapore

**Keywords:** hepatocellular carcinoma, systemic treatment, multi-targeted kinase inhibitors, immune checkpoint inhibitors, antiangiogenic agents

## Abstract

**Simple Summary:**

For years, the systemic therapies sorafenib and lenvatinib have represented standard of care for first-line treatment of advanced hepatocellular carcinoma (HCC). The recent approval of atezolizumab in combination with bevacizumab heralded the arrival of immunotherapy for first-line treatment of advanced HCC, and the field is growing, with other combination immunotherapies under investigation. Focusing on the Asia–Pacific region, where drug availability and reimbursement systems differ widely, this article reviews the evolving treatment landscape and summarises the authors’ expert opinion on therapeutic decision-making to optimise outcomes in advanced HCC.

**Abstract:**

Hepatocellular carcinoma (HCC) is the fourth most common driver of cancer-related death globally, with an estimated 72% of cases in Asia. For more than a decade, first-line systemic treatments for advanced or unresectable HCC were limited to the multi-targeted kinase inhibitors sorafenib and, more recently, lenvatinib. Now, treatment options have expanded to include immunotherapy, as exemplified by the immune checkpoint inhibitor (ICI) atezolizumab combined with the antiangiogenic agent bevacizumab. Additional combinations of ICIs with kinase inhibitors, other ICIs, or antiangiogenic agents are under investigation, further supporting the new era of immunotherapy for first-line treatment of advanced or unresectable HCC. We describe this evolving landscape and provide expert opinion on therapeutic best practices in the Asia–Pacific region, where different costs of, and patient access to, treatment are a challenge. With the combination of atezolizumab plus bevacizumab likely to become the clinical standard of care, optimising treatment sequence and ensuring patient access to newer therapies remain priorities. Cost containment and treatment sequencing may be facilitated by characterisation of predictive positive and negative biomarkers. With these considerations in mind, this review and expert opinion focused on advanced HCC in the Asia–Pacific region offers perspectives of multiple stakeholders, including physicians, payer systems, and patients.

## 1. Introduction

Liver cancer is a common cancer in multiple geographically diverse regions, especially East Asia (an area that includes China, Japan, South Korea, and Taiwan) and Southeast Asia (which includes Singapore) [1]. One of the highest incidence rates of liver cancer worldwide is in Asia [2], and the highest number of cases is in China, due to a high incidence rate (18.3 per 100,000) and the world’s largest population (1.4 billion persons) [1,2]. Hepatocellular carcinoma (HCC), comprising 75% to 85% of primary liver cancer cases [1], is the fourth most common cause of cancer-related death worldwide [3]. An estimated 72% of cases are in Asia [4].

In Asia, diverse systems are used to stage HCC and, although the Barcelona Clinic Liver Cancer (BCLC) staging system is widely used to compare treatment outcomes, it is less widely used to decide management of HCC. Management is guided in Japan by the Japanese Integrated Staging system and in Taiwan by a modified version of the Union for International Cancer Control system, but the BCLC staging system serves as a complementary system there [5]. Like the BCLC system, the Hong Kong Liver Cancer staging system provides treatment guidance as well as prognostic classification [3]. Here, for ease of comparison and discussion of treatment approaches, we refer to the BCLC system, which classifies patients according to tumour status, liver function (by Child–Pugh (CP) category), and performance status (PS) as defined by the Eastern Cooperative Oncology Group (ECOG) [6].

In the BCLC system, stage B (intermediate) HCC is treated with locoregional therapy, the most common of which is transarterial chemoembolisation (TACE), and stage C defines “advanced” HCC, for which the standard of care is palliative systemic therapy [7]. Patients with BCLC-C HCC have cancer-related symptoms, macrovascular invasion, or extrahepatic spread [6]. The term “advanced HCC” can have different meanings, however. In Hong Kong and in Singapore, HCC with macrovascular invasion but without extrahepatic spread is referred to as “locally advanced HCC”. “Advanced HCC” may also define the population of patients for whom systemic therapies are indicated, including those with TACE-refractory BCLC-B HCC. Here, for simplicity, we use “advanced HCC” to refer to BCLC-C HCC. Since 2007, the standard of care for the first-line systemic treatment of advanced HCC has been sorafenib; in 2018, another orally administered, molecular-targeted agent, lenvatinib, was shown to be non-inferior to sorafenib. Both agents inhibit multiple tyrosine kinases [8] and are widely approved in Asia–Pacific countries and territories.

Now, with multiple systemic agents in development, the treatment landscape is expanding. In the phase 3 study of first-line combination therapy with the programmed cell-death 1 ligand 1 (PDL1) inhibitor atezolizumab and the vascular endothelial growth factor (VEGF) inhibitor bevacizumab versus standard-of-care sorafenib for advanced HCC (IMbrave150 trial, NCT03434379), treatment with the novel combination resulted in significantly better outcomes than were achieved with sorafenib [9]. Such an expansion of options brings with it the challenges of treatment selection and sequence. A further challenge is presented by the substantially different healthcare systems and reimbursement criteria in effect in the politically and culturally diverse Asia–Pacific region. Addressing these challenges requires a holistic approach that considers all stakeholders—from patients and healthcare practitioners to the government agencies responsible for approval and reimbursement decisions. With that in mind, and in light of positive results of combination immunotherapy from the IMbrave150 trial, we, as representatives of several key constituents of the Asia–Pacific region, seek to describe the current and evolving treatment landscapes in advanced HCC with the aim of providing expert opinion on best practices for treatment of advanced HCC in our region, with special focus on China, Japan, Singapore, South Korea, and Taiwan.

## 2. Current and Emerging Systemic Therapies in Advanced HCC

Multiple HCC treatment guidelines are relevant in the Asia–Pacific region [5,10,11,12,13,14,15,16,17], and these guidelines continue to evolve as new therapies emerge. Current Asian guidelines for treatment of advanced HCC are summarised in Table 1 [12]. The Pan-Asian adapted European Society for Medical Oncology guidelines, which reflect many other guidelines in the management of advanced HCC, state that “sorafenib is the standard of care for patients with advanced HCC and those with intermediate-stage (BCLC B) disease not eligible for, or progressing despite, locoregional therapies. It is recommended in patients with well-preserved liver function and ECOG PS 0–2” [12]. Additionally, “lenvatinib showed non-inferiority in terms of efficacy compared with sorafenib and can be considered in patients with advanced HCC without main portal vein invasion and with ECOG PS 0–1 as a front-line systemic treatment” [12].

Approval of sorafenib was based on the results of the SHARP trial, conducted primarily in Europe and the USA, [18] and a second trial of almost identical design conducted primarily in an Asia–Pacific population [19]. Median overall survival (OS) was, in the SHARP trial, 10.7 months in the sorafenib group versus 7.9 months in the placebo group (hazard ratio (HR) 0.69, 95% confidence interval (CI) 0.55–0.87), and in the Asia–Pacific trial, 6.5 versus 4.2 months (HR 0.68, 95% CI 0.50–0.93). Grade 3 or 4 adverse events (AEs) occurring with sorafenib were hand–foot skin reaction, diarrhoea, and weight loss (in both trials and in post-marketing registries) [20]. Both sorafenib trials enrolled only patients with CP-A liver status, but post-marketing data suggest that sorafenib has a similar safety and tolerability profile in CP-B patients, and a recent meta-analysis showed that CP-B liver function is associated with a shorter median OS (4.6 months versus 8.8 months for CP-A patients) [20,21].

In the REFLECT trial, lenvatinib was shown to be non-inferior to sorafenib in terms of median OS (13.6 versus 12.3 months; HR 0.92, 95% CI 0.79–1.06), the primary endpoint [22]. Lenvatinib was significantly better (*p* < 0.0001) than sorafenib in several secondary endpoints defined by the modified Response Evaluation Criteria in Solid Tumors (mRECIST): progression-free survival (PFS) (median 7.4 versus 3.7 months; HR 0.66, 95% CI 0.57–0.77), time to progression (median 8.9 versus 3.7 months; HR 0.63, 95% CI 0.53–0.73), and objective response rate (24.1% versus 9.2%; odds ratio 3.1, 95% CI 2.2–4.6) [22]. The incidence of AEs was similar overall in the two treatment groups, but the specific AEs differed: grade ≥3 hand–foot skin reaction occurred in 11% of the sorafenib group but only 3% of the lenvatinib group, whereas grade ≥3 hypertension was more common in the lenvatinib group (23%) than in the sorafenib group (14%), as were grade ≥3 proteinuria (6% versus 2%, respectively) and anorexia (5% versus 1%, respectively) [20,22].

**Figure 1:**
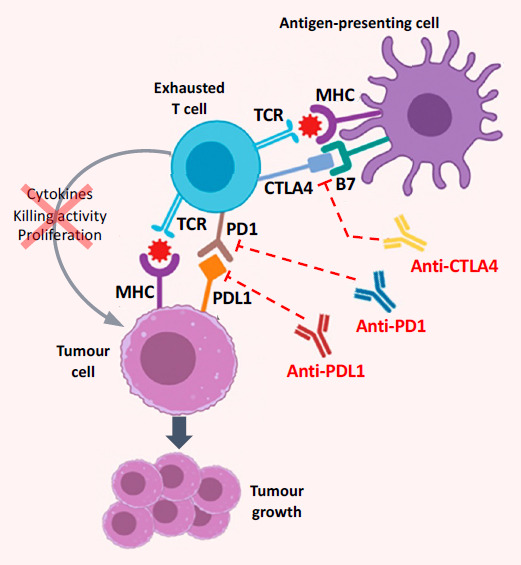
Mechanism of action of immune checkpoint inhibitors (antibodies that target CTLA4, PD1, or PDL1) in the HCC tumour microenvironment, where chronic inflammation and cirrhosis lead to immune exhaustion. Exhausted T cells have a reduced capacity to produce cytokines, to proliferate, and to kill tumour cells; tumour growth is facilitated by blockade of signalling resulting from interaction of the T-cell receptor (TCR) with the major histocompatibility complex (MHC). Key actionable drivers of immune exhaustion in HCC are the PD1–PDL1 pathway and CTLA4 signalling, and blockade of these pathways (i.e., immune checkpoint inhibition) enhances the immune reaction against the tumour cells [32,33]. (Adapted from Giannini et al. 2019 [33].)

Sorafenib is the only first-line systemic therapy for advanced HCC for which subsequent second-line targeted agents are approved. These second-line agents include the multikinase inhibitors regorafenib and cabozantinib; ramucirumab, an antibody targeting the VEGF receptor; nivolumab, pembrolizumab, and camrelizumab, three immune checkpoint inhibitors targeting programmed cell-death protein 1 (PD1); and nivolumab with ipilimumab, an antibody targeting cytotoxic T-lymphocyte-associated protein 4 (CTLA4) (combination approved only in the USA [23]) [3,8,24,25,26]. The mechanism of action of the three immune-checkpoint inhibitors is shown schematically in Figure 1. The current approval and reimbursement status of first- and second-line systemic therapies for advanced HCC in areas of the Asia–Pacific region is shown in Table 2. This class of agents is expanding, and multiple targeted agents are in late-phase clinical development for use in first line in regimens based on combinations of kinase inhibitors, anti-VEGF agents, and immune checkpoint inhibitors (Table 3) [20,27,28]. Promising preliminary results have been presented on two combination regimens: one of the two checkpoint inhibitors durvalumab (which targets PDL1) and tremelimumab (which targets CTLA4) (Study 22, NCT02519348) [29], the other, of the immune checkpoint inhibitor pembrolizumab (which targets PD1) and the kinase inhibitor lenvatinib (LEAP-002 trial, NCT03713593) [30,31].

In the IMbrave150 trial [9], at the primary analysis (29 August 2019 cut-off date) median OS was 13.2 months with sorafenib but not reached with atezolizumab + bevacizumab; the HR was 0.58 (95% CI 0.42–0.79) (*p* < 0.001). Median PFS (95% CI) per RECIST v1.1 by Independent Review Facility (IRF) was 4.3 (4.0–5.6) months with sorafenib and 6.8 (5.7–8.3) months with atezolizumab + bevacizumab (HR 0.59, 95% CI 0.47–0.76; *p* < 0.001), and the overall response rates (per IRF RECIST v1.1) were 12% and 27%, respectively (*p* < 0.001). AE rates were similar in the two treatment groups (grade 3 or 4, 55% versus 57%; grade 5, 6% versus 5%, respectively). Compared with sorafenib, atezolizumab + bevacizumab provided clinically meaningful benefits in key aspects of the patient experience (quality of life, functioning, key symptoms), and atezolizumab + bevacizumab was approved first by the US Food and Drug Administration (FDA) on 29 May 2020 for the treatment of unresectable or metastatic HCC in patients who have not received prior systemic therapy. The combination subsequently received European Commission approval on 2 November 2020, following a positive opinion from the European Medicines Agency’s Committee for Medicinal Products for Human Use on 17 September 2020, for the treatment of advanced or unresectable HCC in adult patients who have not received prior systemic therapy. As of 1 March 2021, marketing authorisation approval has been granted in various Asia–Pacific territories including (in alphabetical order) Australia, Brunei, China, Hong Kong, Japan, Malaysia, Myanmar, New Zealand, Pakistan, Philippines, Singapore, South Korea, Taiwan, and Thailand. Importantly, at the follow-up analysis after an additional 12 months (31 August 2020 cut-off date), the median OS was 19.2 months with atezolizumab plus bevacizumab versus 13.4 months with sorafenib (HR 0.66, 95% CI 0.52–0.85; *p* = 0.0009), a result that validated the clinically meaningful treatment benefit of the combination therapy [34].

These marketing authorisation approvals reflect the safety and efficacy outcomes of the IMbrave150 trial. Although these outcomes were favourable, establishing the atezolizumab + bevacizumab combination as standard of care in any single healthcare system will depend on access, which in turn is determined by pricing and reimbursement policy. In some parts of the Asia–Pacific region, reimbursement criteria are highly complex [12]. In much of the region, ongoing healthcare reforms to achieve universal health coverage mean that healthcare systems and access policies are in a state of flux [35,36], and only relatively recently has health technology assessment (HTA) begun to inform pricing and reimbursement decisions [37]. This dynamic and complex situation only adds to the challenges of introducing a new therapy.

## 3. Healthcare and Reimbursement Systems in the Asia–Pacific Region

In Asia, as in much of the rest of the world, HTA agencies are increasingly responsible for assessing new drugs and making reimbursement recommendations based on cost-effectiveness and budget impact [37,38]. Access can be problematic if cost-effectiveness and budget impact are not satisfactory. Throughout the region, availability and reimbursement may be facilitated by data on biomarkers or other subgroup identification criteria that define patients who are or are not likely to benefit from the treatment. Uniquely, in Singapore, approval of a new drug rapidly follows its approval by the US FDA, and Singapore has the quickest approval process in Asia. Prescribing practice in Singapore follows global guidelines, but in the absence of rigid guidelines, physicians are free to choose treatments—including off-label uses—and to do so on the basis of positive phase 3 trial data, if the drug is accessible.

A key aspect of any reimbursement system is the extent to which it allows access to patients’ therapy of choice. In Japan, most individuals are covered by national health insurance or social (or employees’) health insurance, under which patients pay 10–30% of medical costs (depending on age and income) [37]. In China, 95.1% of the total population had health insurance coverage by 2013 [35]; coverage should now be universal [37]. Patients pay 10–20% of the cost of drugs on the Chinese National Reimbursement Drug List [39]. In 2009, Korean National Health Insurance reimbursed approximately 80% of covered inpatient care and 50–70% of covered outpatient care. Reimbursement policy in Taiwan is similar to that in South Korea. In Singapore, healthcare costs are covered by nationalised life insurance schemes and deductions from compulsory savings plans; levels of governmental subsidies vary [12]. Although selected older cancer drugs are partially subsidised, newer drugs are currently not reimbursed, but Singaporeans with personal medical insurance are likely to have coverage for these drugs.

It is in the context of these heterogeneous healthcare and reimbursement systems, and their responses to evolving standards of care in advanced HCC, that we provide the following expert opinion on approaches to management of this disease in our region.

## 4. Expert Opinion: Treating Advanced HCC in the Asia–Pacific Region

### 4.1. Sorafenib and Lenvatinib Remain First-Line Treatment Choices

On the basis of the patient population enrolled in the IMbrave150 study, it appears that for patients with advanced HCC for whom immunotherapy is not appropriate, such as those at higher risk (poor liver function, immune deficiency, or prior transplantation), sorafenib and lenvatinib remain treatments of choice until further clinical trial and real-world evidence is obtained for immunotherapy approaches. Both drugs are approved in much of the Asia–Pacific region, where they are widely reimbursed as first-line therapy. Lenvatinib has become more commonly used in China and, unsurprisingly, in Japan—the home of its manufacturer. Although in Japan 80% of patients receive lenvatinib in first line, the lack of validated second-line agents after lenvatinib creates challenges for sequencing strategy, and some physicians advocate using sorafenib in first line because another molecular-targeted agent, regorafenib, has been validated for use in second line after failure of sorafenib. Lenvatinib is therefore sometimes reserved for use in third line [40]. In South Korea, some physicians or patients may reject lenvatinib as first-line therapy because second-line therapy after progression on lenvatinib is not reimbursed, unlike second-line regorafenib after progression on sorafenib.

Although no significant difference in OS benefit has been shown between sorafenib and lenvatinib, in trials or in real-world clinical practice [41], lenvatinib may be preferred for antitumour efficacy, as defined by response rates and PFS, and for tolerability (hypertension with lenvatinib versus hand–foot skin reaction with sorafenib). Although the occurrence of hand–foot skin reaction appears to be associated with better survival [42,43], the quality of life of patients experiencing this AE may be significantly impaired; physicians observe that hand–foot skin reaction can be a barrier to the acceptance of sorafenib by some patients. The notoriety of this AE reflects the longevity of use of sorafenib and the communication, throughout its period of use, of anecdotal information among patients and physicians. We find that both sorafenib and lenvatinib demonstrate manageable toxicity profiles and recommend in all settings that physicians and care teams provide comprehensive and balanced information to patients to select treatment of choice.

In our opinion, patients with tumour-related symptoms or a high tumour volume, for whom tumour shrinkage is important, should be offered lenvatinib, even if they narrowly miss the strict inclusion criteria of the REFLECT trial. This opinion is supported by the REFLECT trial finding that in several mRECIST-defined endpoints (mentioned above), lenvatinib was superior to sorafenib as shown by, respectively, a median PFS of 7.4 versus 3.7 months, a median time to progression of 8.9 versus 3.7 months, and an objective response rate of 24.1% versus 9.2% [22]. Real-world data are needed to identify this borderline population. It is the assessment of the authors that sorafenib and lenvatinib should both be offered to BCLC-B and BCLC-C HCC patients, and although supporting data are lacking, we believe, on the basis of our own clinical experience, that lenvatinib should not be withheld from patients with main portal vein invasion as long as they have good liver function (CP A). On the basis of real-world data from the observational REFINE study, sorafenib followed by regorafenib may be preferred for patients with CP-B (possibly even CP-C) liver function [44,45,46].

Another important population of patients to consider is that with advanced HCC of viral aetiology. Globally, 80% of HCC cases are accounted for by chronic HBV or HCV infection [3]. HBV-related HCC predominates in China (where 85% of patients have HBV-related HCC [47]), South Korea (70–80% in the author’s institution), and Singapore (just over 50% in the authors’ institutions), in contrast to Japan, where (in the author’s institution) 50% of HCC cases are of non-viral aetiology, 40% are HCV related, and only 10% are HBV related. Thanks to Japan’s surveillance programme for HBV and HCV infection, HCC of viral aetiology is detected early and treated with locoregional therapies or resection, and HCC patients in Japan have the longest OS worldwide [48]. The predominant aetiology of HCC in Japan has changed dramatically in recent decades: HCC of non-viral aetiology has increased from 10.0% in 1991 to 32.5% in 2015 [49]. As there is no surveillance for non-viral HCC, patients present with more advanced disease and are then more likely to receive systemic therapy. In Singapore, too, the distribution of aetiologies has changed in the past 5–10 years, with HBV-related HCC becoming less common and non-viral HCC more common [50,51]. Therefore, the impact of HCC of viral aetiology is significant, but there are insufficient data to support the view that the response to systemic therapy differs according to molecular target or to drive selection of therapy for advanced HCC. The idea that efficacy differs by viral aetiology currently applies to sorafenib versus lenvatinib, but at present there is no direct evidence supporting this hypothesis: the data that suggest a survival benefit with sorafenib in HBV-negative, HCV-positive patients are from subgroup and post hoc analyses [52,53,54] and, therefore, require validation by further investigation.

### 4.2. The IMbrave150 Trial Results Are Practice-Changing

On the basis of the efficacy and safety profiles of atezolizumab + bevacizumab versus sorafenib in the first-line treatment of advanced HCC, this combination therapy is likely to become the standard of care in the Asia–Pacific region. This opinion is reflected in marketing authorisation approval granted, within a few months of approval by the US FDA, in more than 10 Asia–Pacific territories (as listed above). Publication of the IMbrave150 trial’s results has already led to updating of local guidelines. For example, following a consensus meeting the Taiwan Liver Cancer Association updated its guidance to include the atezolizumab + bevacizumab combination for the treatment of unresectable HCC in patients who have not received prior systemic therapy and do not have a high risk of upper gastrointestinal bleeding [16].

Given that at the initial analysis (clinical data cut-off date 29 August 2019, median follow-up duration 8.6 months) median OS had not been reached in the atezolizumab + bevacizumab arm (versus 13.2 months, 95% CI 10.4—not evaluable in the sorafenib arm) [9], the earliest marketing authorisations and guideline updates were made on the basis of a perceived (rather than proven) efficacy of the atezolizumab + bevacizumab combination. However, these decisions were subsequently validated by the later analysis (31 August 2020 cut-off date) in which the median OS was 19.2 months with atezolizumab + bevacizumab versus 13.4 months with sorafenib (HR 0.66, 95% CI 0.52–0.85; *p* = 0.0009) [34]. We recognise that assessment of the validity and impact of OS data in clinical studies in advanced HCC may be confounded by length of follow-up and the use of second-line or later therapy, especially immuno-oncologic-based therapies. In the era of immune checkpoint inhibitors, more data are needed on alternative endpoints such as PFS, which might offer the benefit of trial completion in less time and, therefore, at lower cost. However, OS and PFS data together provide a better picture of efficacy, and until other endpoints are validated, OS remains the gold standard endpoint in clinical trials of 2–3 years’ duration for first- and second-line treatments for advanced HCC. Although sufficient follow-up duration is crucial, median OS is an essential indicator of absolute benefit that is informative for physicians and patients alike.

In addition to survival data, patient-reported outcomes assessed in the IMbrave150 trial adequately reflect patients’ experiences and have real-world validity. As clinical trial data mature and show different treatments to have similar efficacy, the patient experience, expressed by patient-reported outcomes, will become increasingly important. The IMbrave150 study used both disease-specific and general patient-reported outcomes. In calculating QALYs it is best to use patient-reported outcomes of similar type; disease-specific patient-reported outcomes may be the better option. One instrument used in the IMbrave150 trial, the EQ-5D (see https://euroqol.org/eq-5d-instruments/, accessed on 30 March 2021), is widely used worldwide and is a commonly accepted utility measure in HTA.

Although the IMbrave150 trial included only a small number of patients with BCLC-B HCC, first-line treatment with atezolizumab + bevacizumab may also be beneficial for patients with TACE-refractory or TACE-unfeasible BCLC-B HCC. In addition, given that the population investigated in IMbrave150 was broader than that in the REFLECT study, the combination may be beneficial for patients with portal vein thrombosis. Although bevacizumab is known to increase bleeding risk, data presented from the IMbrave150 study do not show a clear trend regarding an increased risk of bleeding. Further investigation is needed to identify patients other than those matching the trial population who may benefit. In general, multiple factors should be considered when selecting patients for immuno-oncologic combination therapy. These factors include autoimmune disease status, liver function, the possibility of downstaging to allow resection or transplantation in patients otherwise fit for surgery or transplantation, and prior transplantation.

Another factor influencing the selection of atezolizumab + bevacizumab as first-line therapy for advanced HCC is cost. Although the combination has been approved in more than 10 Asian countries as of 1 March 2021, its regulatory approval does not guarantee accessibility to patients if elevated cost and budget impact (despite good cost-effectiveness) do not allow reimbursement by state or payer systems. Governments in the region must identify drug-pricing strategies that facilitate access while maintaining fiscal responsibility [55]. Being the second largest pharmaceutical market in the region, China—where price–volume agreements and an aggressive tendering process are commonly used to obtain drugs at the lowest possible price—may lead the way [55]. Of note is that cost reduction is anticipated with the availability of biosimilars of bevacizumab, a drug reaching the end of its patent life, along with the price-lowering effect of further treatment combinations becoming available over time. Indeed, promising phase 3 efficacy findings from the phase 2–3 ORIENT-32 study (NCT03794440; see Table 3) of sintilimab combined with a bevacizumab biosimilar as first-line treatment for advanced HCC were presented recently [56]. At data cut-off on 15 August 2020, with a median follow-up of 10.0 months, median OS was significantly longer with the sintilimab plus bevacizumab biosimilar combination (median OS not estimable) than with sorafenib (median OS 10.4 months), and the combination was associated with a 43.1% lower risk of all-cause death (HR 0.569, 95% CI 0.431–0.751; *p* < 0.0001). The combination was also associated with improved PFS (43.5% lower risk of progression as assessed by independent radiologic review committee per RECIST v1.1); median PFS with the combination was 4.6 months, compared with 2.8 months with sorafenib (HR 0.565, 95% CI 0.455–0.701; *p* < 0.0001). These data could provide patients with advanced HCC in China access to a new immunotherapy combination in the first-line setting.

Accessibility to the atezolizumab + bevacizumab combination in the Asia–Pacific region will also depend on the method by which patients pay for treatment (out of pocket, by a reimbursement system, or through healthcare insurance) and will therefore differ throughout the region. For example, it is anticipated that during the time required to establish reimbursement in South Korea (approximately 1–2 years) and in China, patients will pay for the atezolizumab + bevacizumab combination out of pocket. In contrast, in Japan, the combination has been approved and is reimbursed, so cost is not anticipated to be a significant concern for patients. In Singapore, if the combination does not enter the standard formulary, patients will pay out of pocket or through insurance. In this setting, doctors will not prescribe a therapy the patient cannot afford, and for these patients, enrolment in clinical trials or special programmes set up by the pharmaceutical company may improve access.

A more systematic approach to biomarker characterisation in advanced HCC could expedite access by identifying the patients most likely to benefit from a given therapy [57]. Well-characterised and predictive positive and negative biomarkers would permit selection of patients for whom a given therapy will be most effective, or ineffective, thus avoiding the expense of ineffective treatment. One barrier to biomarker characterisation is the difficulty of obtaining tissue biopsy samples: patients who have already undergone multiple therapies may be reluctant to undergo yet another intervention. Nevertheless, biopsy samples should be collected before, during, and after treatment, where possible, and in the context of clinical trials, to expand our understanding of treatment effects on HCC. In the future, liquid biopsy may provide a minimally invasive alternative to tissue biopsy for identifying biomarkers in circulating DNA or RNA and refining HCC prognostication through the use of next-generation sequencing technologies [58,59]. However, further studies and validation of such methods are still required.

The use of biomarkers will also improve our understanding of optimal, and personalised, treatment sequences in advanced HCC. In addition, as demonstrated by Facciorusso et al., identification of prognostic factors to define the best sequence of treatment options will become crucial [60]. As the atezolizumab + bevacizumab combination evolves as first-line treatment of choice and likely standard of care, there comes a need to define second- and later-line options. Although further data are urgently needed to identify optimum treatment sequences, in practice, sorafenib or lenvatinib is likely to be used after the atezolizumab + bevacizumab combination, where possible. This approach has been investigated in a retrospective analysis that demonstrated comparable efficacy as well as manageable toxicities of sorafenib and lenvatinib in patients with advanced HCC after disease progression on atezolizumab + bevacizumab combination therapy [61]. We believe that until results of further studies are available to better define sequential systemic therapy, the agents currently (or formerly) used in second line after sorafenib or lenvatinib (i.e., cabozantinib, nivolumab, pembrolizumab, ramucirumab, regorafenib, and the combination of nivolumab with ipilimumab) will shift to third line. It should be noted that the IMbrave251 study (NCT04770896) is expected to initiate in April 2021 to assess the combination of atezolizumab + lenvatinib or sorafenib versus lenvatinib or sorafenib alone in second line after progression in patients with HCC treated with atezolizumab + bevacizumab. Treatment-sequence decisions are multifactorial even in the absence of biomarker data and integrate physician-driven clinical considerations, drug reimbursement policies, and patient-specific factors such as performance status, liver function, and patient preference.

### 4.3. A Snapshot of Advanced HCC: The Different Perspectives of Patients, Physicians, and Payer Systems

#### 4.3.1. The Patient’s Perspective

For patients with advanced HCC, the priority is delaying disease progression and prolonging life, and patients deserve to be well informed how best to manage their extension of life. Patients need information about holistic factors, including education about the risks of their disease and its treatment, and about practical factors, such as how to optimise supportive care and to manage side-effects. For many, however, a therapy’s safety profile may be weighed against the personal economic burden imposed by it. A patient receiving expensive but non-curative treatment and experiencing significant or debilitating side-effects may forgo treatment, perhaps turning to an alternative (such as traditional herbal medicine, in China).

#### 4.3.2. The Physician’s Perspective

Delaying disease progression is also a priority for the healthcare professional. In the practical clinical experience of the authors, when assessing the anticipated survival benefit of a novel therapy, a reasonable extension of life is represented by a 30% increase in lifespan, or by delay of progression over that obtained with the comparator therapy, that translates to a hazard ratio of 0.7. For a therapy with substantial toxicity, a smaller hazard ratio of 0.5 may make the therapy more acceptable. Such assessments highlight the importance of a multidisciplinary approach in the management of advanced HCC: the tumour management board plays a key role in defining the best treatment plan for each patient. The composition of tumour management boards can differ by institution, but the board should include medical oncology specialists, who have expertise in managing immuno-oncologic therapeutic toxicity.

#### 4.3.3. The Payer System’s Perspective

A balance must be reached between costs (which may include opportunity costs, not just monetary costs) and benefits, as measured in terms of quality-adjusted life-years (QALYs). Another concept common across HTA committees in Asia is the magnitude of benefit relative to the cost—the basis of the incremental cost-effectiveness ratio—which is considered when assessing novel therapies such as the atezolizumab + bevacizumab combination for reimbursement.

## 5. Conclusions

The increasing complexity of therapeutic decision-making for patients with advanced HCC in the Asia–Pacific region reflects not only an expanding range of systemic first-line treatment options but also a variety of healthcare systems, treatment guidelines, and approval patterns in the region. The IMbrave150 study, in which significantly improved survival outcomes were obtained with atezolizumab + bevacizumab versus sorafenib, has revealed an effective new treatment option with the potential to become the standard of care. The clinical need for this novel therapy in the Asia–Pacific region is demonstrated by ongoing regulatory approvals, the onset of reimbursement approvals, and the updating of local guidelines. To help mitigate the cost of therapy to payer systems or to patients themselves, and to improve treatment outcomes, we need to identify the patients who will benefit from the combination, ideally through characterisation of validated prognostic biomarkers. If immunotherapy is not appropriate, sorafenib and lenvatinib remain first-line treatments of choice. As the number of treatment options for advanced HCC increases, treatment sequencing must be optimised to achieve the best possible outcomes for patients. Patients have a fundamental right to understand their disease and their treatment options, and clinicians should provide balanced information not only about treatment efficacy but also about side-effects and their manageability. For patients with HCC, the success of atezolizumab + bevacizumab in prolonging lives in the IMbrave150 trial marks the beginning of a new era and provides a beacon of hope.

## Figures and Tables

**Table 1:** Summary of Asian guidelines for the treatment of advanced (BCLC stage C) HCC with macrovascular invasion or extrahepatic metastases (or both) (adapted and updated from Chen et al. 2020 [12]).

	Country or Region of Origin of the Guideline													
	Asia Pacific (APASL) [11]		China [14]		Hong Kong (HKLCS) [17]		Japan (JSH) [13]		Korea (KLCSG) [5]		Singapore (NCCS) [15]		Taiwan (TLCA) [16]	
**Disease** **features**	MVI^+^	EHM^+^	MVI^+^ (IIIa)	EHM^+^ (IIIb)	Int. I or IIa + Vp 1–3 LA IIb or IIIa + Vp 1–3	EVM (IVa, IVb) Vp 4 or EHM (or both)	MVI^+^	EHM^+^	MVI^+^ (IIc, IIIb, IVa) Single Multiple	EHM^+^ IVa (LN), IVb (others)	MVI^+^	EHM^+^	MVI^+^	EHM^+^
**First-line** **treatment**	Systemic therapy, TACE	CP A or B: systemic therapy CP C: best supportive care	TACE ± (MKI or FOLFOX4, LR, RT)	MKI or FOLFOX4 ± (TACE, RT)	IIb or IIIa, CP A: LR	Systemic therapy	TACE, LR, HAIC, MKI	MKI	TACE (SIRT) ± EBRT Sorafenib Lenvatinib	Sorafenib Lenvatinib	SIRT, TACE	Systemic therapy	LR, MKI	Systemic therapy ± (TACE, SIRT, LR)
**Second-line** **treatment**			MKI	MKI	IIb or IIIa, CP B, or IIIb: TACE				(Vp 1–3: LR x 1–3)	(TACE, EBRT)	Systemic therapy		TACE + RT	Chemo-therapy
**Later line of** **treatment**									(CT, HAIC if MKI failed or not available)				SIRT, HAIC	

APASL, Asian Pacific Association for the Study of the Liver; CP, Child–Pugh; CT, computed tomography; EBRT, external beam radiation therapy; EHM, extrahepatic metastasis; EVM, extrahepatic vascular metastasis; FOLFOX4, folinic acid (leucovorin), 5-fluorouracil, and oxaliplatin; HAIC, hepatic arterial infusion chemotherapy; HKLCS, Hong Kong Liver Cancer Staging system; Int., intermediate; JSH, Japan Society of Hepatology; KLCSG, Korean Liver Cancer Study Group; LA, locally advanced; LN, lymph node; LR, liver resection; MKI, multikinase inhibitor; MVI, macrovascular invasion; RT, radiation therapy; SIRT, selective internal radiotherapy; TACE, transarterial chemoembolisation; TLCA, Taiwan Liver Cancer Association; Vp 1–3, portal vein thrombosis with involvement of unilateral 3rd (Vp1), 2nd (Vp2) or 1st branch (Vp3) of portal vein or bilateral 1st branches.

**Table 2:** Approvals and reimbursements for drugs for the treatment of advanced or unresectable HCC by authors’ country or territory in the Asia–Pacific region (adapted and updated from Chen et al. 2020 [12]).

Country or Territory	First Line			Second Line (After Sorafenib)						
	Sor	Len	Atezo + Bev	Cabo	Rego	Ramu	Nivo	Pembro	Nivo + Ipi	Camre
China	A, R	A, R	A, NR ^a^	NA, NR	A, R	NA, NR	NA, NR	NA, NR	NA, NR	A, R
Japan	A, R	A, R	A, R	A, R	A, R	A, R	NA, NR	NA, NR	NA, NR	NA, NR
Korea	A, R	A, R	A, NR	A, NR	A, R	A, NR	NA, ^b^ NR	NA, NR	NA, NR	NA, NR
Singapore	A, NR	A, NR	A, NR	A, NR	A, NR	A, NR	NA, ^c^ NR	NA, ^c^ NR	NA, NR	NA, NR
Taiwan	A, R	A, R	A, NR	A, NR	A, R	A, NR	A, ^d^ NR	A, ^d^ NR	NA, NR	NA, NR

^a^ Patient access programme available (China Foundation of Cancer; http://www.cfchina.org.cn/show.php?contentid=2192, accessed on 20 January 2021 (in Mandarin)). ^b^ Off-label use is granted by the regulatory agency. ^c^ Readily available for use without approval. ^d^ Only for patients who received approval to use the drug before 1 April 2020 and meet requirements for application for renewal in follow-up evaluation. A, approved; NA, not approved; NR, not reimbursed; R, reimbursed. Atezo, atezolizumab; Bev, bevacizumab; Cabo, cabozantinib; Camre, camrelizumab; Ipi, ipilimumab; Len, lenvatinib; Nivo, nivolumab; Pembro, pembrolizumab; Ramu, ramucirumab; Rego, regorafenib; Sor, sorafenib.

**Table 3:** Ongoing phase 3 clinical trials of new first-line systemic therapy combinations for advanced or unresectable HCC (compiled from information available at www.ClinicalTrials.gov accessed on 25 May 2021 ^a^).

Study Drug (s)	Control Arm	Key Eligibility Criteria	Clinical Trials Identifier (Study Name)	Mechanism of Study Drug	Status
Atezolizumab + bevacizumab	Sorafenib	ECOG PS ≤1, CP A, ≥1 measurable lesion	NCT03434379 (IMbrave150)	Anti-PDL1 Anti-VEGF	Active, not recruiting
Lenvatinib + pembrolizumab	Lenvatinib + placebo	ECOG PS ≤1, BCLC stage B or C, CP A, ≥1 measurable lesion	NCT03713593 (LEAP-002)	MKI Anti-PD1	Active, not recruiting
Sintilimab + bevacizumab biosimilar IBI305	Sorafenib	ECOG PS ≤1, BCLC stage B or C, CP score ≤7, ≥1 measurable lesion	NCT03794440 (ORIENT-32)	Anti-PD1 Anti-VEGF	Active, not recruiting
Cabozantinib + atezolizumab	Sorafenib	ECOG PS ≤1, BCLC stage B or C, CP A, measurable disease	NCT03755791 (COSMIC-312)	Anti-VEGFR Anti-PDL1	Recruiting
Camrelizumab (SHR-1210) + apatinib	Sorafenib	ECOG PS ≤1, BCLC stage B or C, CP A, ≥1 measurable lesion	NCT03764293	Anti-PD1 Anti-VEGFR2	Recruiting
Camrelizumab (SHR-1210) + FOLFOX4	Placebo + FOLFOX4	ECOG PS ≤1, CP score ≤7, measurable disease	NCT03605706	Anti-PD1 Chemotherapy	Recruiting
Durvalumab ± tremelimumab	Sorafenib	ECOG PS ≤1, BCLC stage B or C, CP A	NCT03298451 (HIMALAYA)	Anti-PDL1 Anti-CTLA4	Recruiting
IBI310 + sintilimab	Sorafenib	ECOG PS ≤1, BCLC stage B or C, CP score ≤6, ≥1 measurable lesion	NCT04720716	Anti-CTLA4 Anti-PD1	Recruiting
Lenvatinib ± CS1003	Placebo	ECOG PS ≤1, BCLC stage B or C, CP A, ≥1 measurable lesion	NCT04194775	MKI Anti-PD1	Recruiting
Nivolumab + ipilimumab	Sorafenib or lenvatinib	ECOG PS ≤1, CP A, ≥1 measurable lesion	NCT04039607 (CheckMate 9DW)	Anti-PD1 Anti-CTLA4	Recruiting
SCT-I10A + bevacizumab biosimilar SCT-510	Sorafenib	ECOG PS ≤1, BCLC stage B or C, CP score ≤7, ≥1 measurable lesion	NCT04560894	Anti-PD1 Anti-VEGF	Recruiting
HLX10 + HLX04	Sorafenib	BCLC stage B or C, ≥1 measurable lesion	NCT04465734	Anti-PD1 Anti-VEGF	Not yet recruiting
Penpulimab injection + anlotinib	Sorafenib	ECOG PS ≤1, BCLC stage B or C, CP score ≤7, ≥1 measurable lesion	NCT04344158	Anti-PD1 MKI	Not yet recruiting

^a^ The following search terms were entered into the “Advanced Search” field at www.ClinicalTrials.gov, accessed on 25 May 2021: “Condition or disease: HCC”, “Other terms: advanced”, “Study type: Interventional Studies (Clinical Trials)”; recruitment status was selected as “Recruiting”, “Not yet recruiting” and “Active, not recruiting”; and filters were applied to select phase 3 and industry-funded studies. The search results were reduced to a list of only trials of first-line systemic combination therapy in patients with advanced or unresectable HCC. BCLC, Barcelona Clinic Liver Cancer; CTLA4, cytotoxic T-lymphocyte-associated protein 4; CP, Child–Pugh; ECOG, Eastern Cooperative Oncology Group; HCC, hepatocellular carcinoma; MKI, multiple kinase inhibitor; PD1, programmed cell death 1; PDL1, programmed death-ligand 1; PS, performance status; VEGF(R), vascular endothelial growth factor (receptor).
